# Digital product success under the microscope: When artificial intelligence in projects helps — and when it hurts

**DOI:** 10.1371/journal.pone.0331229

**Published:** 2025-08-29

**Authors:** Mina Nikolić, Dragan Bjelica

**Affiliations:** Department of Management and Project Management, Faculty of Organizational Sciences, University of Belgrade, Belgrade, Serbia; Ataturk University, TÜRKIYE

## Abstract

As organizations navigate an increasingly dynamic digital landscape, the challenge of achieving consistent product success has intensified. This study investigates how key management factors—customer-driven product development, open innovation networks, organizational digital agility, and AI-integrated project management—influence digital product outcomes. Special attention is given to the dual role of artificial intelligence: as a potential enabler of innovation and a possible constraint when applied in rigid or misaligned ways. A quantitative survey was conducted among 239 professionals engaged in product-related roles across diverse industries and regions. Data were analyzed using linear regression, moderation analysis, and non-parametric testing to assess both direct and interaction effects among the variables. The results reveal that customer-driven product development, open innovation networks, and organizational digital agility each have a statistically significant positive impact on product success, with customer-driven development emerging as the strongest predictor. In contrast, AI-integrated project management does not demonstrate a significant direct effect. Notably, AI negatively moderates the relationship between open innovation networks and product success, suggesting that while AI may enhance structured knowledge-sharing, it can also diminish the creative and collaborative elements essential for innovation if not carefully managed. These findings highlight the strategic complexity of integrating AI into digital product development. While AI can enhance operational efficiency and knowledge flows, its impact on innovation outcomes is context-dependent and may disrupt the balance between human creativity and automated decision-making. The study underscores the need for hybrid models in which AI complements—not replaces—human expertise. Insights from this research offer valuable guidance for organizations aiming to design resilient, customer-centric, and innovation-driven digital product strategies in an AI-enhanced environment.

## Introduction

As digital transformation accelerates, organizations face mounting pressure to deliver innovative, high-performing digital products that can adapt to fast-changing market demands. Yet, despite advancements in technology and methodology, achieving digital product success remains a formidable challenge. Businesses today must not only generate bold ideas but also master the intricate alignment of customer-centric strategies, open collaboration, organizational agility, and increasingly, artificial intelligence (AI).

While decades of research have highlighted the importance of customer insights, cross-functional teamwork, and agile organizational structures [1–3], the expanding role of AI introduces new opportunities—and uncertainties—into the equation [4]. As a tool for automation, data analysis, and intelligent decision-making, AI is increasingly reshaping how digital products are conceived, developed, and delivered [5]. However, its broader impact on innovation dynamics, team collaboration, and long-term product outcomes remains complex and context-dependent. In many cases, the integration of AI is still evolving, and its potential is misunderstood, particularly when applied without alignment to human-centered and adaptive innovation practices [4,5].

Compounding this complexity are the persistent practical challenges faced by organizations integrating AI into their operations. According to MuleSoft’s 2025 Connectivity Benchmark Report, top barriers include outdated IT infrastructure (41%), cybersecurity risks (41%), and a shortage of specialist AI talent (38%). Other concerns such as data quality issues (33%), compliance risks (34%), and ethical dilemmas (27%) reflect the broader technical, legal, and organizational hurdles of adopting AI at scale. Strikingly, only 3% of organizations report experiencing no barriers when applying data to AI use cases—underscoring just how widespread these challenges remain [6]. Similarly, IBM’s 2024 AI Adoption Challenges report highlights several critical obstacles to AI adoption. Notably, 45% of organizations express concerns about data accuracy or bias, while 42% cite insufficient proprietary data to customize models effectively. Additionally, 42% report inadequate generative AI expertise as a significant barrier [7]. These findings emphasize the multifaceted challenges organizations face in integrating AI technologies into their operations.

In light of these sobering figures, it becomes clear that the path to digital product success is not only technical but deeply strategic. This study seeks to explore the interplay between Customer-Driven Product Development, Open Innovation Networks, Organizational Digital Agility, and AI-Integrated Project Management, asking a critical question: Does AI enhance the product innovation process—or inadvertently constrain it? By empirically investigating the direct and moderating effects of AI on these key drivers of success, this research offers a timely contribution to the ongoing conversation on how organizations can build smarter, more resilient digital products in an AI-enhanced world.

## Literature review

### Determinants of digital product success

Digital product success depends on customer-driven development because it aligns products with market needs which drives innovation, performance improvements and at the same time reduces exposure to development risk. Organizations achieve higher adoption rates and stronger value propositions when they incorporate customer feedback and insights into their product development process to create products that better meet consumer demands [1,8]. Active customer participation in the development process encourages greater innovation, as user-generated insights drive the creation of new product features and solutions that may not emerge solely from internal teams [9]. Moreover, customer involvement enhances product performance by refining functionality, usability, and overall quality, though its success is contingent upon technological capabilities and the stage of the development process [10,11]. Beyond innovation benefits, customer-driven product development also contributes to cost efficiency and risk reduction by allowing firms to eliminate unnecessary features early in development, leading to more efficient resource allocation, as previously reported [12]. Additionally, companies that effectively manage customer knowledge and engagement gain a competitive advantage, strengthening customer relationships and brand loyalty while ensuring market-aligned product offerings [1]. However, while customer-driven product development is widely recognized as a strategic approach for aligning innovation with user needs, recent literature cautions that excessive reliance on customer input may constrain breakthrough innovation. Some studies find that customer involvement often leads to incremental improvements rather than radical product shifts, especially when customers lack the vision for unarticulated or disruptive needs [13]. Moreover, the effectiveness of customer-driven strategies can vary across industries and organizational maturity levels [10]. These unresolved tensions point to a need for a more contextual understanding of CDPD, particularly when paired with other organizational capabilities like digital agility and AI-driven project practices. This study contributes to this debate by exploring how customer-centricity interacts with broader innovation frameworks in shaping digital product outcomes.

Beyond customer involvement, digital product success depends on open innovation which is a paradigm that suggests organizations can and should use external as well as internal ideas and market pathways to enhance their technology. This approach contrasts with the traditional model of innovation, where companies rely solely on their internal research and development. Open innovation improves digital product success through enhanced innovation performance and product development speed while building digital trust and promoting sustainability with economic growth. Open innovation enables organizations to develop advanced digital technologies through the integration of multiple external knowledge sources and resources which are vital for continuous innovation [1,14,15]. Deep external collaborative efforts alongside broad partnerships help accelerate new product development when they match an efficient business model [16]. Open innovation strategies become more effective through the enhancement of digital trust which results from Industry 4.0 technology adoption and this leads to innovation processes and products that deliver higher efficiency and impact [17]. Moreover, open innovation partnerships between firms and universities, NGOs and industry stakeholders create pathways to economic success and sustainability objectives which show that open innovation systems can support both environmental responsibility and profitability [18]. On the other hand, emerging research highlights key limitations. Overextending openness can lead to challenges such as IP leakage, reduced strategic focus, increased coordination complexity, and difficulty in managing trust across boundaries [19,20]. Some scholars argue for an inverted U-shaped relationship, where moderate openness maximizes performance, while excessive collaboration may hinder it [19]. This debate remains unresolved, particularly in digital contexts where collaboration is often mediated by AI systems. This study addresses this gap by examining how AI influences the relationship between open innovation and product success, providing evidence that openness may become less effective when mediated by rigid or overly structured AI-driven project systems.

Another important factor for product success in digital environments is digital agility which helps organizations to quickly adjust to disruptions, increase customer responsiveness and integrate digital resilience and workforce agility. When firms can quickly react to emerging digital changes they find new opportunities for innovation which keeps them competitive in rapidly changing markets [2,21]. Companies use data analytics and aggregation tools to boost customer agility which enables them to detect and react to customer preference shifts and market trends essential for achieving new product success [22]. Organizational agility together with information systems agility makes digital product development successful by enabling firms to seize digital opportunities more quickly than their competitors which proves crucial in converging markets [23]. The combination of collaborative mindsets with innovation capabilities and adaptability in workforce agility significantly advances digital transformation and drives product innovation. Organizations become more resilient when employees engage in knowledge sharing and continuous learning, as reported in recent analysis [24]. Digital agility enables small and medium-sized enterprises (SMEs) to take risks and adapt, which proves essential for managing digital turbulence while achieving innovative performance [25,26]. Organizations maintain long-term product success through digital resilience which includes digital adaptation together with cybersecurity preparedness and operational continuity to withstand disruptions. Businesses that integrate strategic agility with digital platform capabilities can better respond to unstable conditions while improving their market stance and product outcomes [27]. However, recent work suggests that agility alone does not guarantee performance. In some cases, agility may emerge as a reactive response to environmental volatility rather than a proactive strategic asset [28]. Furthermore, the capability to pivot quickly can strain organizational coherence and lead to fragmentation in decision-making [29]. These nuances suggest that digital agility must be studied in combination with complementary factors such as innovation networks, customer engagement, and AI-enabled processes. The present study contributes by positioning digital agility within a broader framework of enablers and testing its conditional impact on digital product success.

### The role of AI in digital product management

Artificial intelligence (AI) is becoming more and more ingrained in digital product development; consequently, both in terms of practical impact and theoretical grounding, its influence on project workflows, decision-making, and innovation results requires closer attention. The interplay between AI, innovation processes, and organizational contexts can be better understood through three complementary theoretical perspectives: the Technology-Organization-Environment (TOE) framework, the Diffusion of Innovations (DOI) theory, and the Socio-Technical Systems (STS) theory.

The TOE framework asserts that an organization’s technology adoption is shaped by three domains: the technological context (e.g., the availability and capability of AI tools), the organizational context (e.g., internal agility, structure, and culture), and the external environment (e.g., collaboration networks, market demands) [30]. This model directly corresponds with the constructs examined in this study, encompassing AI-integrated project management, digital agility, and open innovation networks.

The DOI theory explains how innovations like AI diffuse through organizations based on perceived attributes such as relative advantage, complexity, compatibility, and trialability [31]. These characteristics help interpret AI’s dual role—where it can support knowledge structuring and decision-making, but also hinder creativity and collaboration if perceived as too rigid or misaligned with innovation processes.

Complementing these two models, the Socio-Technical Systems (STS) theory emphasizes that successful technology implementation depends on the joint optimization of technical systems (e.g., AI platforms) and social systems (e.g., people, roles, workflows) [32]. When these two subsystems are misaligned, performance and innovation suffer. STS theory helps explain why AI might weaken open innovation outcomes: if AI structures override informal collaboration channels or suppress human creativity, the social dimension of innovation becomes constrained. In digital product management, balancing algorithmic efficiency with human flexibility is essential—a principle rooted in the STS perspective.

Collectively, these frameworks provide a systematic perspective for examining the facilitators, limitations, and interaction effects influencing AI’s impact on the development of digital products. They direct the formulation of our hypotheses and elucidate the interpretation of empirical results in AI-augmented contexts.

#### AI-integrated project management: benefits vs. challenges.

The management of digital products is being transformed by AI as it optimizes each phase of the product lifecycle from ideation to market launch to increase efficiency and competitiveness. AI-driven tools in market research and validation provide data-driven decision-making capabilities by analyzing consumer trends, preferences, and sentiments which support product strategy [5,33]. Through its ability to adapt based on user feedback and market demands AI speeds up the prototyping process and design iterations in addition to making products more adaptable and enhancing user experience via personalized interfaces [5,34]. Furthermore, AI-powered automation enhances quality assurance and testing operations which leads to increased product reliability and reduced failure risks [5]. AI enhances product launch marketing techniques with predictive analytics and targeted advertising and at the same time adjusts pricing dynamically to boost market penetration together with consumer engagement [5,35]. The success of AI implementations in product management requires both strong digital infrastructure and effective data integration to enable intelligent decision-making processes and enhance business resilience [36,37]. Product managers who use AI to work across these areas achieve more efficient development processes while enhancing user satisfaction and staying competitive in dynamic markets.

On the other hand, AI faces critical limitations in digital product management, particularly in unstructured knowledge-sharing, creativity, and adaptability, which impact decision-making and innovation. Processing unstructured data remains a challenge, as AI relies on rule-based methods that struggle with complex document formats and real-time analysis, making it difficult to extract actionable insights [38]. AI systems can enable knowledge sharing but require strong organizational structures and clear data-sharing rules to protect privacy which restricts their capability to enhance cross-functional teamwork as demonstrated in prior studies [39,40]. In driving innovation, AI supports creativity but lacks independent ideation, requiring structured processes and alignment with broader sustainability and innovation strategies [39,41]. Adaptability is another key constraint, as AI systems struggle with sudden market shifts and disruptions, such as the COVID-19 pandemic, where predictive models fail due to non-representative data [42]. Also, even with techniques like transfer learning, AI can only adapt effectively when historical data aligns with emerging patterns [42]. The current restrictions necessitate ongoing refinement of AI product management approaches through enhanced human supervision and agile methodologies along with better data governance to fully realize AI’s potential in digital product success.

#### AI as a moderator: enhancing or weakening innovation?.

Research indicates that AI improves digital product management collaboration through structured knowledge-sharing facilitation and the optimization of organizational knowledge management processes. Furthermore, studies suggest that AI can support knowledge workers by redesigning roles and workflows, allowing for more efficient information exchange and learning [43,44]. AI-powered tools may help structure unstructured data, identify relevant insights, and provide real-time access to critical information for product teams. Research recommends customizing AI systems for knowledge work because they demonstrate the critical need for a balance between human skills and automated processes [45]. The integration of AI into team-based structures is expected to advance cross-departmental dialogue while speeding up resolution mechanisms and enhancing product development workflows.

However, some studies indicate that AI creates decision-making obstacles within open innovation networks and digital product management because it establishes fixed structures and prompts ethical and privacy issues while requiring human supervision. Decision-making systems powered by AI rely heavily on data-driven approaches that reduce their ability to adapt creatively to new innovations [4,46,47]. AI systems operate under strict guidelines because their design prioritizes efficiency and consistency which can limit creativity and flexible methods in digital product development and collaborative innovation processes [48,49]. Additionally, the integration of AI into decision-making processes introduces ethical concerns, particularly regarding data privacy and bias, which can further complicate innovation initiatives [47,49]. To meet organizational objectives while fulfilling ethical standards organizations must implement additional human supervision of AI decision-making processes. Without appropriate oversight, AI may lead to decisions that are technically optimal but misaligned with broader innovation objectives [45,47].

Challenges in AI-generated knowledge further complicate its role in open innovation and digital product management. AI systems, particularly generative models, can introduce biases and lead to the misapplication of knowledge. The prior research papers document a tendency for models to generate central data distribution outputs that lead to a detrimental “knowledge collapse” phenomenon where increased information access harms public understanding [50,51]. This bias can impact open innovation networks by reinforcing mainstream ideas while suppressing emerging perspectives and reducing the diversity of insights that drive breakthrough innovations. Additionally, the application of AI for content creation introduces ethical and privacy challenges because AI-generated content (AIGC) presents security threats that require regulatory solutions such as watermarking to safeguard intellectual property against unauthorized distribution [52]. The heavy dependence on AI for knowledge transfer and application introduces dangers by sidelining new knowledge workers and decreasing human participation in innovation processes which results in diminished knowledge exchange within digital product ecosystems [50,51]. Without strategies to mitigate these challenges, AI may inadvertently constrain the effectiveness of open innovation networks and digital product management rather than enhancing them.

While AI excels in automating repetitive tasks and improving efficiency in digital product management, some studies suggest that this focus on optimization can overshadow the inimitable qualities of human creativity and tacit knowledge [53]. AI systems face difficulties in understanding creativity’s complex and ambiguous characteristics which are essential for developing groundbreaking innovations and solving open-ended challenges. Open innovation networks generate new ideas through varied human interactions and chance discoveries and yet they risk stifling radical innovation when they become too dependent on AI-driven efficiency [53]. Organizations need to maintain a balance between AI-driven automation and human creativity to ensure digital product ecosystems retain their innovative and exploratory qualities which algorithms cannot replace.

## Methodology

This study employed a quantitative, survey-based research approach to collect data from respondents. The survey consisted of two sections: socio-demographic questions to capture participants’ background characteristics and questions measuring key constructs within the research model, including Open Innovation Networks, AI-Integrated Project Management, Organizational Digital Agility, Customer-Driven Product Development, and Product Success. Google Forms served as the platform for survey administration which provided both accessibility and efficient data collection.

Following data collection, responses were analyzed using SPSS 30.0 to conduct descriptive statistics, linear regression analysis, moderation analysis, and non-parametric testing. These quantitative statistical methods were applied to test the proposed hypotheses and explore the relationships among the constructs.

### Research scope and hypotheses

This study aimed to analyze the impact of Customer-Driven Product Development along with Open Innovation Networks, Organizational Digital Agility and AI-Integrated Project Management on Digital Product Success (H1). The evolving nature of digital markets demands organizations to integrate customer insights (H1a), leverage external knowledge-sharing (H1b), and maintain digital agility (H1c) in order to achieve long-term product success [2,8,54]. However, the expanding use of AI in project management still leaves the nature of its impact on digital product innovation uncertain [41]. Despite AI being recognized for improving decision-making and operational efficiency it remains unclear how it impacts innovation results specifically within product development frameworks [55]. Given that the direct effect of AI on Digital Product Success remains unclear (H1d), further analysis was required.

Building on these foundational relationships, this study further investigates whether AI-Integrated Project Management moderates the influence of Open Innovation Networks, Organizational Digital Agility, and Customer-Driven Product Development on Digital Product Success (H2, H3, H4). By assessing whether AI strengthens or weakens these relationships, the study provides insights into whether AI serves as an enhancer of knowledge-sharing and innovation efficiency or if its rigid decision-making processes and automation-driven approaches introduce challenges that may limit its potential. To further investigate these dynamics, non-parametric test was conducted to examine how AI adoption impacts knowledge-sharing practices within Open Innovation Networks (H5). The goal was to determine whether higher AI usage fosters more structured knowledge exchange or whether its influence alters the quality and effectiveness of shared knowledge in digital innovation ecosystems.

These findings contribute to the broader understanding of AI’s evolving role in project management, open collaboration, and digital product success in an era of increasing technological reliance. A summary of all research hypotheses is presented in Table 1.

**Table 1:** Research hypotheses.

H#	Hypothesis Statement
H1	Customer-Driven Product Development, Open Innovation Networks, Organizational Digital Agility, and AI-Integrated Project Management significantly contribute to explaining Digital Product Success.
H1a	Customer-Driven Product Development has a positive effect on Digital Product Success.
H1b	Open Innovation Networks have a positive effect on Digital Product Success.
H1c	Organizational Digital Agility has a positive effect on Digital Product Success.
H1d	AI-Integrated Project Management has a positive effect on Digital Product Success.
H2	AI-Integrated Project Management moderates the relationship between Open Innovation Networks and Digital Product Success.
H3	AI-Integrated Project Management moderates the relationship between Customer-Driven Product Development and Digital Product Success.
H4	AI-Integrated Project Management moderates the relationship between Organizational Digital Agility and Digital Product Success.
H5	Knowledge-sharing practices differ significantly across varying levels of AI adoption in project management.

**Fig 1:**
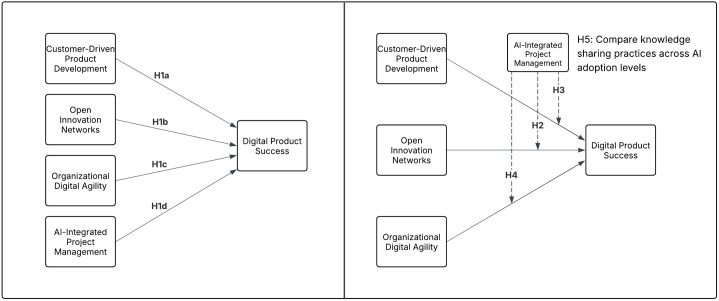
Visual representation of hypotheses.

To support the hypotheses outlined in Table 1, Fig 1 presents a visual representation of the research model. It illustrates the direct effects of the key management factors on Digital Product Success, the moderating role of AI-Integrated Project Management, and also mentions the comparison of knowledge-sharing practices across different levels of AI adoption

### Research procedure

**Fig 2:**
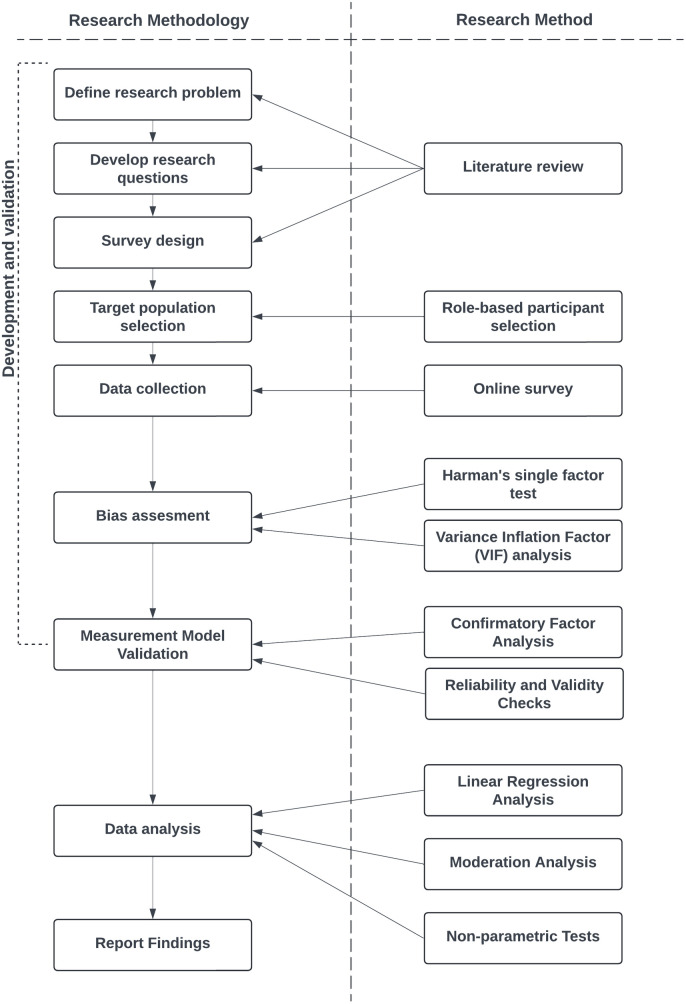
Research procedure.

The research methodology of this study was designed to systematically investigate the factors contributing to Digital Product Success. The procedure followed several structured steps to ensure the reliability and validity of the data, aligned with the research procedure illustrated in Fig 2.

The research process began with defining the research problem, which was informed by an extensive literature review. The review established a theoretical base to explore essential concepts including Customer-Driven Product Development together with Open Innovation Networks, Organizational Digital Agility, and AI-Integrated Project Management. Research questions emerged from previous studies which directed their construction to match existing theoretical frameworks and empirical results.

After establishing research questions a survey design was created to collect relevant data for the study’s specific constructs. The questionnaire was carefully structured to ensure clarity and reliability, with survey questions developed based on a thorough review of existing literature to ensure strong content validity. The target population selection process focused on professionals working in product-related roles, such as product managers, product owners, business development managers and other product related roles. A role-based participant selection approach was employed to ensure responses were gathered from individuals with direct experience in digital product development. The survey was disseminated via professional communities, LinkedIn groups, and targeted outreach to product managers across various industries specializing in digital product development. The data collection phase included administering the survey online via Google Forms, providing convenience and accessibility for respondents. All survey questions were mandatory to maintain data integrity which minimized missing data and produced a full dataset for analysis.

To address potential common method bias, Harman’s single-factor test was conducted using exploratory factor analysis with principal axis factoring to determine whether a single factor accounted for the majority of variance. Additionally, a full collinearity assessment was performed by calculating Variance Inflation Factor (VIF) values for all model constructs, serving as an alternative diagnostic for method bias in single-source survey data. To validate the measurement model, a Confirmatory Factor Analysis (CFA) was carried out using Smart PLS v4.1.1.4. Construct reliability and convergent validity were assessed through Cronbach’s alpha, Composite Reliability (CR), and Average Variance Extracted (AVE). Discriminant validity was further evaluated using the Fornell-Larcker criterion.

The data analysis phase encompassed multiple statistical techniques. Linear regression analysis was used to assess the direct effects of the independent variables on Digital Product Success. Additionally, moderation analysis was conducted to determine whether AI-Integrated Project Management influenced the strength of these relationships. Finally, given the observed interaction effects, nonparametric test (Kruskal-Wallis test) was performed to explore variations in knowledge-sharing practices across different levels of AI adoption in project management.

The final phase involved reporting findings based on the results of the statistical tests. These results provided valuable insights into the factors influencing Digital Product Success, contributing to academic knowledge and offering practical implications for industry professionals, policymakers, and researchers.

By following this structured procedure, the study ensured rigorous methodology, robust data integrity, and meaningful contributions to the discourse on Digital Product Success in AI-enhanced environments.

### Measurement items

Each independent construct in the model was operationalized as a single latent variable, measured as the average of 12 survey items spanning three theoretically grounded subdimensions. All items were rated on a 5-point Likert scale (1 – Strongly Disagree, 5 – Strongly Agree). This composite measurement approach provides a more comprehensive representation of each construct, capturing both the breadth and depth of the underlying concept. The development of survey items was firmly grounded in established literature, following recommended practices for content validity and scale construction [56]. Items were adapted from previously validated sources and tailored to the context of digital product development and management, ensuring conceptual accuracy and contextual relevance. To enhance clarity and alignment with professional terminology, the survey instrument was iteratively reviewed by internal experts in product development. Although no formal pilot study was conducted, this expert review process served as a quality checkpoint for refining item wording and improving construct fit. The complete set of survey questions is provided in the supplementary material as S2 File, while a detailed appendix outlining the operationalization of constructs and corresponding literature sources is available as S3 File.

Open Innovation Networks were measured as the average of Knowledge Co-Creation, Knowledge Sharing and Distribution, and Collaborative Technologies Integration. Items for Knowledge Co-Creation were adapted from open innovation framework [57] and studies on collaboration [58], emphasizing external partnerships and their role in driving innovation. Measures for Knowledge Sharing and Distribution were informed by research on social networks [59] and review on organizational collaboration [60], highlighting the significance of knowledge dissemination and collective intelligence in innovation processes. Lastly, the integration of Collaborative Technologies was measured using items derived from research on digital innovation ecosystems [61] and study on interorganizational relationships [62], focusing on the technological enablers of collaboration and productivity.

AI-Integrated Project Management was measured as the average of AI Utilization, AI Effectiveness, and AI Integration. Items for AI Utilization were informed by research on IT success [63] and work on AI challenges and opportunities [64], assessing the extent to which AI tools are integrated into project workflows. Measures of AI Effectiveness leveraged insights from study on analytics capabilities [65] and exploration of AI in data-driven decision-making, capturing AI’s role in enhancing planning, risk assessment, and project performance [66]. Finally, AI Integration was measured using items inspired by work on enterprise AI applications [67] and research on AI-driven business model innovations [68], evaluating the degree to which AI contributes to efficiency and project outcomes.

Organizational Digital Agility was measured as the average of Digital Adaptation, Cybersecurity and Risk Management, and Digital Recovery and Continuity. Digital Adaptation was assessed using items from review of digital transformation [69] and study on digital strategy [70], reflecting organizational adaptability to technological changes. Measures of Cybersecurity and Risk Management were informed by research on cybersecurity governance [71] and study on enterprise risk management [72], ensuring the scale captured organizational preparedness for digital threats. Digital Recovery and Continuity items were adapted from business continuity framework [73] and handbook on disaster recovery [74], emphasizing the role of resilience in sustaining digital operations.

Customer-Driven Product Development was measured as the average of Customer Involvement in Innovation, Market Research and Analysis, and Product Customization and Personalization. Customer Involvement in Innovation was captured using measures based on meta-analysis of customer engagement [75] and co-creation framework [76], highlighting the active role of customers in shaping product development. Market Research and Analysis measures were derived from research on marketing strategy effectiveness [77] and work on customer insights [78], reflecting the importance of continuous market feedback in guiding product innovation. Product Customization and Personalization was assessed using scales validated by study on customer value creation [79] and updated Technology Readiness Index [80], evaluating the extent to which product development aligns with customer needs and preferences.

Digital Product Success was measured as the average of Product Performance, Market Performance, and Innovation and Quality. Product Performance was assessed using measures from research on new product development drivers [8] and study on customer experience management [81], ensuring that the scale captured customer satisfaction, usability, and functionality. Market Performance was measured using items adapted from findings on sustainable product development [82] and research on profitability and customer satisfaction metrics [83], reflecting market competitiveness and commercial success. Lastly, Innovation and Quality was measured using items from meta-analytic review on firm innovativeness [84] and research on supply chain integration [85], ensuring that the scale accounted for product differentiation, technological advancement, and overall quality standards.

By measuring each construct as the average of multiple validated dimensions, this study ensures high reliability, internal consistency, and theoretical validity in assessing the factors contributing to Digital Product Success.

### Data collection and sample description

The survey distribution was restricted to professionals with product-related positions such as product managers and product owners together with other essential digital product development roles to maintain data reliability and relevance. The targeted sampling approach produced a respondent pool with adequate expertise and experience for delivering meaningful insights on digital product success. The survey was disseminated through prominent professional groups and communities specializing in product management and digital innovation. Specifically, it was shared among members of the Serbian Product Community, Product Managers Community, Digital Product Managers, Product Owner’s Help Desk, and the Product Development and Management Association (PDMA). The survey was distributed through these carefully selected professional communities relevant to product management and digital innovation, ensuring that it reached a knowledgeable and well-defined audience. To enhance the reliability and validity of the collected data, the sampling strategy targeted participants from diverse industries, company sizes, and geographic regions, thereby reducing selection bias. This purposive yet varied approach aimed to capture a broad spectrum of perspectives applicable to digital product development and innovation. Although the survey was shared within several large professional communities, the total number of individuals exposed to it through these channels is unknown, making it impossible to determine an exact response rate for this segment of the distribution.

The survey was conducted anonymously between June 12th and September 1st, 2024. Participants were informed on the first page of the survey that participation was voluntary, responses would remain anonymous, and the study aimed to examine how different management practices influence product success. Informed consent was obtained in the form of implied consent—by proceeding with the survey, participants acknowledged this information and agreed to participate. No written or verbal consent was collected, as the study involved no interventions, no collection of personal or sensitive data, and no inclusion of vulnerable populations. Given the anonymous and minimal-risk nature of the study, ethics approval was not required.

The final dataset comprised 239 fully completed responses, with no missing data. Female participants made up the majority at 55.2%, while males accounted for 43.9%, and 0.8% chose not to specify their gender. The educational profile of the sample was notably high, with 47.3% possessing a bachelor’s degree and 41.4% holding a master’s degree. A significant portion of respondents (40.6%) reported more than a decade of professional experience. In terms of company size, 38.5% were employed in organizations with 51–200 employees, and 21.8% worked in firms with 201–500 employees. The geographical distribution showed that most participants were affiliated with companies located in Europe (54.4%), followed by South America (20.5%), North America (17.6%), and Asia (7.5%). A detailed breakdown of these characteristics is available in Table 2.

**Table 2:** Sample demographics (N = 239).

Characteristic variables	Value	Frequency	Percentage(%)
Gender	Male	105	43.9
	Female	132	55.2
	Prefer not to say	2	0.8
Education	High school or equivalent	20	8.4
	Bachelor’s degree	113	47.3
	Master’s degree	99	41.4
	Doctorate (PhD)	6	2.5
	Other	1	0.4
Experience	Less than 1 year	20	8.4
	1-3 years	22	9.2
	4-6 years	41	17.2
	7-10 years	59	24.7
	More than 10 years	97	40.6
Company Size	11-50 employees	21	8.8
	51-200 employees	92	38.5
	201-500 employees	52	21.8
	501-1000 employees	31	13.0
	More than 1000 employees	43	18.0
Main Company Location	North America	42	17.6
	South America	49	20.5
	Europe	130	54.4
	Asia	18	7.5

Despite the diversity of the sample across sectors and regions, the predominance of European respondents may introduce a degree of regional bias. The high representation from Europe could reflect context-specific trends in digital innovation, shaped by regionally tailored policies and market conditions, which may limit the extrapolation of findings on a global scale. From an organizational perspective, the sample skews toward professionals in mid-sized companies, though nearly one-third (31%) are drawn from larger enterprises with over 500 employees. This distribution aligns well with environments where structured innovation processes and digital strategies are more formally embedded, yet may not fully capture the dynamics of smaller, early-stage startups with leaner operations and more fluid roles. However, the sample’s substantial professional experience—particularly the 65% who have spent over seven years in product-related positions—and high academic qualifications lend credibility to the study. These attributes suggest that participants are likely to have meaningful involvement in product innovation and decision-making processes. To broaden the applicability of the findings, future studies might consider comparative research designs that account for regional, cultural, and organizational differences.

### Assessment of common method bias

Harman’s Single-Factor Test was performed to assess the existence of common method bias. The exploratory factor analysis (EFA) test uses principal axis factoring to assess the variance that one factor explains. When a single factor explains a large portion of the variance, the study results may reflect measurement artifacts instead of genuine variable relationships. Analysis results reveal that the initial factor explains 43.614% of the total variance which falls short of the 50% standard threshold established in research [86]. The absence of dominant variance by any single factor indicates that common method bias does not pose a significant problem in this dataset.

To further address concerns related to potential bias from single-source data collection, we conducted a full collinearity assessment using Variance Inflation Factor (VIF) analysis in SPSS. While VIF primarily detects multicollinearity, it is also considered a proxy for common method bias in regression frameworks [87]. All VIF values for the independent variables in the model ranged from 1.409 to 2.582, well below the recommended conservative threshold of 3.3. These results suggest that common method bias is unlikely to significantly distort the observed relationships among the variables.

By confirming that the data is not excessively influenced by the measurement method, this test supports the validity of the findings. The absence of substantial common method bias ensures that the observed relationships between Open Innovation Networks, AI-Integrated Project Management, Organizational Digital Agility, Customer-Driven Product Development, and Digital Product Success are likely reflective of genuine effects rather than artifacts of the survey methodology.

### Measurement model assessment

Confirmatory Factor Analysis (CFA) was first conducted using Smart PLS v4.1.1.4 to evaluate the overall fit of the measurement model. The results indicated a strong model fit, with χ²/df = 2.98, CFI = 0.949, TLI = 0.933, and SRMR = 0.041. These values meet commonly accepted thresholds for good model fit—χ²/df below 3.0, CFI and TLI above 0.90, and SRMR below 0.08—confirming that the observed data adequately reflect the proposed latent structure [88]. These indices provide strong support for the construct validity of the measurement model and justify further assessment of reliability and convergent validity.

Construct reliability and convergent validity were assessed using Cronbach’s alpha, Composite Reliability (CR), and Average Variance Extracted (AVE). Cronbach’s alpha is a widely used measure of internal consistency, with values above 0.70 considered acceptable [89,90]. All five main constructs demonstrated strong internal consistency, with alpha values ranging from 0.847 (ODA) to 0.937 (DPS). To further assess internal consistency, Composite Reliability was calculated. CR provides a more robust estimate than Cronbach’s alpha, as it does not assume equal factor loadings and better accounts for the congeneric nature of measurement scales [91]. All constructs exceeded the 0.70 threshold, with CR values between 0.858 and 0.937.

Convergent validity was evaluated using AVE, which reflects the average variance explained by a construct’s indicators. AVE values above 0.50 indicate adequate convergent validity [91]. In this model, AVE ranged from 0.669 to 0.833, confirming that each construct captures a substantial portion of variance in its items. A summary of Cronbach’s alpha, CR, and AVE for each main construct is presented in Table 3.

**Table 3:** Construct reliability and convergent validity results.

Construct	Cronbach’s Alpha	CR	AVE
Open Innovation Networks	0.865	0.863	0.685
AI-Integrated Project Management	0.876	0.878	0.703
Organizational Digital Agility	0.847	0.858	0.669
Customer-Driven Product Development	0.916	0.919	0.793
Digital Product Success	0.937	0.937	0.833

Additionally, discriminant validity was assessed using the Fornell-Larcker criterion. For most construct pairs, the square root of AVE exceeded the inter-construct correlations, confirming adequate discriminant validity. A marginal violation was observed between Customer-Driven Product Development (CDPD) and Digital Product Success (DPS), where the inter-construct correlation slightly exceeded the square root of AVE. However, the constructs are theoretically distinct but practically interrelated, as customer-driven development efforts often directly contribute to product outcomes. As noted by Henseler et al., such overlaps are acceptable when constructs serve different theoretical roles but are closely linked in practice, particularly when all other validity criteria are satisfied [92].

## Results

### Regression model

The model summary indicates strong explanatory power because the R² value of 0.804 demonstrates that four independent variables explain 80.4% of the variance in Product Success. The model demonstrates strong predictive capability with an Adjusted R² value of 0.801 when accounting for the number of predictors. Statistical significance was assessed at the 0.05 level (p < .05). Accordingly, the ANOVA results confirmed statistical significance (F = 240.196, p < 0.001) indicating that at least one independent variable significantly affects Product Success prediction. These results provide strong empirical support for hypothesis H1, affirming that the identified management factors influence Product Success.

**Fig 3:**
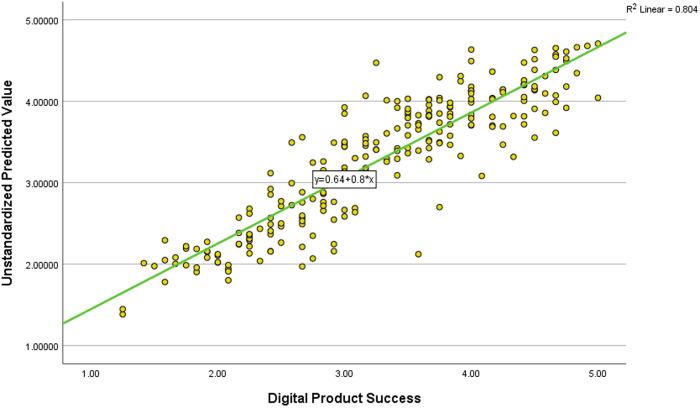
Regression Model Fit: Predicted vs. Actual Digital Product Success.

Additionally, Fig 3 presents a scatterplot of unstandardized predicted values and observed values for Product Success, illustrating a strong linear relationship. The fitted regression line, with an R² value of 0.804, reinforces the model’s robustness in explaining Product Success. The clustering of data points around the regression line further indicates a strong positive correlation, supporting the model’s validity.

#### Impact of individual variables on product success.

When examining the influence of individual variables in the model on Product Success, Customer-Driven Product Development stands out as the most significant predictor. Research findings demonstrate that companies which focus on synchronizing product development efforts with customer requirements and feedback achieve the greatest positive effect on Product Success (B = 0.586, β = 0.636, p < 0.001). The standardized coefficient value of 0.636 demonstrates that Customer-Driven Product Development is the independent variable with the highest contribution to Product Success variance which validates hypothesis H1a.

The Open Innovation Networks demonstrated a strong positive effect on Product Success evidenced by B = 0.166 and β = 0.179 with statistical significance p < 0.001. The standardized coefficient (β = 0.179) reveals that organizations achieve better product success when they actively engage in knowledge-sharing and external collaboration. The significance of this variable reaffirms its contribution to Product Success and validates hypothesis H1b.

Similar to the previous variable, Organizational Digital Agility showed a substantial positive effect on Product Success which was indicated by B = 0.146 and β = 0.145 with p-value less than 0.001. The standardized coefficient (β = 0.145) demonstrates that organizations which exhibit strong digital adaptability alongside efficient technology integration and digital transformation navigation experience higher product success. The statistical significance of this variable verifies its fundamental role in Product Success thus affirming hypothesis H1c.

Interestingly, the use of AI-Integrated Project Management did not show a significant direct effect on Product Success compared to other independent variables (B = 0.073, β = 0.067, p = 0.051). The AI impact on Product Success remains inconclusive at the conventional significance level (p < 0.05) yet its marginal statistical probability (p = 0.051) indicates potential context-dependent effects or the presence of other influencing factors. To test for a potential nonlinear relationship, a quadratic regression was conducted using both AI-Integrated Project Management and its squared term (AI²) as predictors of Product Success. The results showed that the linear term was statistically significant (β = .698, p = .043), while the squared term was not (β = −0.254, p = .461), indicating that the relationship between AI and product success does not follow a U-shaped or inverted-U pattern. This suggests that AI’s influence on product outcomes is better captured through linear or conditional interaction effects. As a result, hypothesis H1d is rejected. The regression coefficients for all independent variables, including their respective significance levels and effect sizes, are summarized in Table 4.

**Table 4:** Regression coefficients table.

Predictor Variable	B	Std. Error	Beta	t	Sig.
Open Innovation Networks	.166	.040	.179	4.162	< 0.001
AI-Integrated Project Management	.073	.037	.067	1.964	.051
Organizational Digital Agility	.146	.038	.145	3.826	< 0.001
Customer-Driven Product Development	.586	.043	.636	13.678	< 0.001

The marginal significance emphasizes the need for additional research into how AI-Integrated Project Management moderates the interactions between Open Innovation Networks and both Organizational Digital Agility and Customer-Driven Product Development to understand Product Success. Examining AI’s moderating effects can provide deeper insights into whether AI strengthens or weakens the impact of these key management factors on product success.

Given the high proportion of European respondents in the sample (54.4%), we conducted a robustness check using only non-European data (n = 109) to assess the generalizability of our findings. The core pattern remained consistent: Customer-Driven Product Development remained the strongest predictor of Product Success (β = 0.646, p < .001), followed by Open Innovation Networks (β = 0.177, p < .001) and Organizational Digital Agility (β = 0.137, p = .001), both showing significant positive effects. AI-Integrated Project Management remained non-significant (β = 0.064, p = .115). These results suggest that the primary relationships identified in this study extend beyond the European context.

To examine potential variation in digital product success across industry settings, we conducted subgroup linear regression analyses comparing professionals from the IT industry (n = 139) and those from non-IT industries (n = 100). Both models were statistically significant and explained a substantial proportion of variance in product success (Adjusted R²_IT = 0.794; Adjusted R²_non-IT = 0.809). Customer-Driven Product Development consistently emerged as the strongest predictor across both groups (β_IT = 0.556, p < 0.001; β_non-IT = 0.690, p < 0.001). Organizational Digital Agility was significant in both subgroups, with nearly identical effect sizes (β_IT = 0.141, p = 0.009; β_non-IT = 0.146, p = 0.012). Open Innovation Networks also demonstrated significance in both settings, showing a stronger effect in the IT industry (β_IT = 0.255, p = 0.001) compared to the non-IT group (β_non-IT = 0.140, p = 0.013). In contrast, AI-Integrated Project Management did not exhibit a statistically significant effect in either subgroup (p > 0.05). These findings suggest that the primary drivers of digital product success are consistent across industries, while the impact of AI appears to be more limited and context-dependent.

### Moderation analysis

To further examine the role of AI-Integrated Project Management, moderation analysis was conducted to determine whether AI influences the relationships between Open Innovation Networks, Organizational Digital Agility, and Customer-Driven Product Development with Product Success. The moderation effects were assessed using interaction terms in the regression models.

The interaction terms for AI-Integrated Project Management with Organizational Digital Agility (Aavg × Davg) and Customer-Driven Product Development (Aavg × Cavg) did not yield statistically significant effects on Product Success. The influence of AI on how Organizational Digital Agility affects Product Success proved statistically negligible (B = 0.007, β = 0.034, p = 0.919) which shows that incorporating AI does not change the way digital agility affects product results. Similarly, the combined effect of AI-Integrated Project Management with Customer-Driven Product Development did not achieve statistical significance with results of B = −0.061 and β = −0.298 at p = 0.084. Since AI-Integrated Project Management did not significantly moderate the effects of Organizational Digital Agility and Customer-Driven Product Development, attention is directed toward its moderating effect on the Open Innovation Networks, which yielded statistically significant results.

Unlike the other interactions, the AI-Integrated Project Management × Open Innovation Networks (Aavg × Kavg) interaction term was found to be statistically significant and negative (B = −0.144, β = −0.693, p < 0.001). This result suggests that AI-Integrated Project Management weakens the positive relationship between Open Innovation Networks and Product Success. While Open Innovation Networks independently contribute to higher Product Success, the presence of AI in project management reduces this positive effect.

To further investigate this relationship, a correlation analysis was conducted between AI-Integrated Project Management and the Open Innovation Network to examine their direct association. Additionally, a Kruskal-Wallis test evaluated if knowledge-sharing approaches vary with different levels of daily AI application in project management. The supplementary analysis provides enhanced understanding about the effects of AI integration on knowledge-sharing processes and its impact on product success.

**Fig 4:**
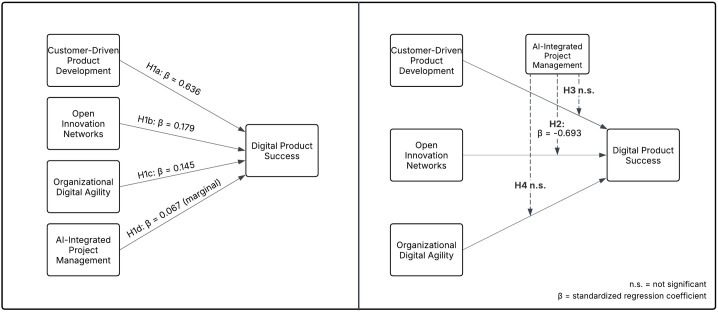
Hypothesis model with regression and moderation outcomes.

To consolidate the results of both the regression and moderation analyses, Fig 4 presents the research model with standardized regression coefficients.

#### Correlation and group differences in knowledge sharing based on AI usage.

The Pearson correlation coefficient between AI-Integrated Project Management (Aavg) and Open Innovation Network (Kavg) is 0.339, indicating a moderate positive correlation (p < 0.001). This suggests that organizations that integrate AI into project management tend to have higher levels of collaborative knowledge-sharing practices. The correlation reaches statistical significance at the 99% confidence level because the significance value (p < 0.001) falls well below the 0.01 threshold.

**Fig 5:**
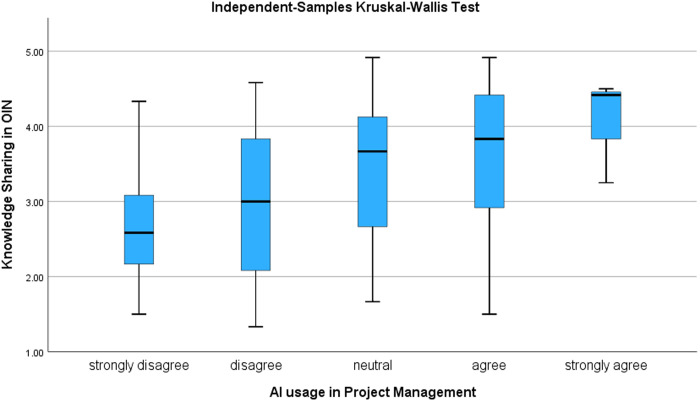
Variation in knowledge sharing across AI usage levels in project management.

The Kruskal-Wallis test results provide additional support for the correlation by analyzing how knowledge-sharing practices differ with various daily AI usage levels in project management. The test results were statistically significant (H = 26.298, p < 0.001), leading to the rejection of the null hypothesis that knowledge-sharing practices are uniformly distributed across AI usage levels. The accompanying boxplot, presented in Fig 5, illustrates a clear trend—higher AI usage in project management correlates with greater engagement in knowledge-sharing practices, further validating the positive relationship observed in the correlation analysis.

Together, these results indicate that AI usage significantly influences knowledge-sharing behaviors in project environments. While greater AI adoption fosters more structured, intensive, and systematic knowledge exchange, the moderation analysis reveals that this enhanced collaboration does not always lead to higher product success. This highlights the complex role of AI-Integrated Project Management and sets the stage for its conceptual interpretation, which is further explored in the Discussion.

## Discussion

### Interpretation of regression results

This study demonstrates that Customer-Driven Product Development, Open Innovation Networks, and Organizational Digital Agility significantly contribute to Digital Product Success, collectively explaining 80.4% of its variance. The high explanatory power underscores these management strategies as critical drivers of digital product performance. The strong overall model significance (p < 0.001) reinforces the importance of integrating strategic knowledge-sharing, customer engagement, and digital adaptability to achieve success in today’s competitive digital markets.

Among the examined factors, Customer-Driven Product Development emerged as the strongest predictor. This highlights the imperative for organizations to align product development strategies with user needs and expectations. Prior research supports the effectiveness of agile methodologies, user-centered design, and co-creation in improving product adoption and long-term market performance [93]. Companies that systematically incorporate customer feedback into innovation workflows enhance usability, increase engagement, and improve product-market fit.

Similarly, Open Innovation Networks were shown to positively influence product success. By leveraging external partnerships and collaborative ecosystems, firms gain access to diverse expertise and advanced technologies that accelerate innovation and enrich digital offerings [94]. Especially in fast-paced environments, the ability to integrate external knowledge serves as a key competitive advantage.

Organizational Digital Agility also demonstrated a significant effect on product success. Firms with strong technological adaptability and resilient digital infrastructure are better equipped to respond to market shifts, integrate emerging technologies, and sustain innovation over time [95]. These results align with previous findings that emphasize digital agility as a core enabler of successful digital transformation.

In contrast, AI-Integrated Project Management did not show a statistically significant direct effect on product success (p = 0.051), although the result approached the threshold for marginal significance. Research shows that while AI is generally seen as transformative for project management and strategic planning the success of AI depends on factors such as implementation quality and human-AI collaboration [96]. This near-significant result does not suggest irrelevance, but rather points to the conditional nature of AI’s impact. Supporting this, our findings reveal a positive correlation between AI usage and knowledge-sharing practices within organizations, indicating that AI can strengthen the foundational mechanisms for innovation. However, the negative moderation observed between AI and open innovation suggests that in some contexts, AI may constrain rather than enhance collaborative innovation—particularly when it introduces excessive structure or reduces flexibility. The marginal statistical importance of AI’s direct effect, along with its mixed interaction patterns, highlights the need for further investigation into its indirect and conditional roles within digital product innovation.

In sum, while traditional management factors continue to strongly shape digital product success, the role of AI appears more nuanced and contingent. This calls for future research that moves beyond direct effects and explores how AI interacts with organizational processes, capabilities, and collaboration models in driving innovation performance.

### Moderation analysis: the role of AI-integrated project management

The moderation analysis demonstrates how AI-Integrated Project Management influences complex relationships among Open Innovation Networks, Organizational Digital Agility, and Customer-Driven Product Development to affect Digital Product Success. While AI has been widely considered as an enabler of innovation and efficiency in project management, its moderating effects in this study suggest that its impact may not always be uniformly beneficial.

Specifically, AI-Integrated Project Management showed no significant moderating effect on the relationship between either Organizational Digital Agility or Customer-Driven Product Development and Product Success. This suggests that, in its current form, AI may not substantially alter strategic or customer-facing mechanisms that depend on real-time feedback, iterative learning, and human judgment [24,97]. Many AI tools are designed to automate tasks, optimize resources, or standardize workflows—objectives that do not always align with the dynamic and non-linear nature of agile or customer-centric innovation processes [98]. Previous research also indicates that AI does not necessarily improve decision-making when interpretive flexibility is required [99]. These findings help explain why digital agility and customer-driven practices remain effective independently of AI involvement, and why AI’s moderating role appears limited in these areas.

In contrast, the analysis uncovered a significant negative moderating effect of AI on the relationship between Open Innovation Networks and Product Success (B = −0.144, β = −0.693, p < 0.001). While Open Innovation Networks are generally beneficial, the presence of AI in project management seems to diminish their positive impact. This is a striking result, as it challenges the widespread assumption that AI universally enhances collaborative innovation. One possible explanation is that AI-driven project systems—although efficient at automating and optimizing—may fall short in supporting the flexible, human-centric collaboration that open innovation requires. These systems can impose rigid structures and workflows that suppress tacit knowledge exchange, creative exploration, and informal interaction—factors central to open innovation [51]. Furthermore, when AI systems rely on biased, incomplete, or overly structured data, they can disrupt rather than support innovation outcomes [100]. These risks underscore the need for organizations to ensure that AI supports, rather than replaces, human-led innovation.

This paradox is further illuminated through three theoretical frameworks. The Technology-Organization-Environment (TOE) framework posits that technology adoption is shaped not only by technical capacity but also by organizational readiness and external pressures [30]. Even if organizations are equipped to implement AI, misalignment with internal collaboration structures—especially within open innovation settings—may limit its benefits. Diffusion of Innovations (DOI) theory adds that adoption outcomes depend on perceptions of complexity, compatibility, and advantage [31]; AI may be seen as too rigid or misaligned with exploratory collaboration. Finally, Socio-Technical Systems (STS) theory stresses the need to jointly optimize technical tools and human workflows [32]. If AI is introduced without regard for human context and flexibility, it may suppress rather than support innovation.

Building upon these foundational perspectives, it is also vital to consider the ethical and trust-related dimensions of AI integration, which further explain its paradoxical effects in open innovation settings. While the Socio-Technical Systems theory emphasizes alignment between technology and human workflows, AI governance frameworks [101,102] stress transparency, accountability, and human oversight to prevent algorithmic opacity, bias, or unintended disruption to collaborative values. In parallel, Trust in Automation theory [103,104] suggests that trust in AI must be carefully calibrated through system feedback, predictability, and reliability—especially in innovation environments where cooperation and experimentation are key. To address these concerns, Human-in-the-Loop (HITL) approaches [105,106] advocate for maintaining active human involvement in AI-supported processes to preserve contextual judgment and creativity. Together, these frameworks highlight that AI can enhance knowledge structuring but may also constrain open innovation unless embedded within transparent, trust-centered, and ethically governed systems.

Our correlation and Kruskal-Wallis test results reinforce this interpretation. A statistically significant positive correlation between AI adoption and Open Innovation Networks indicates that organizations using more AI tend to engage more in knowledge sharing. The Kruskal-Wallis test further confirms that knowledge-sharing intensity increases with AI usage. Yet despite this increased structure and activity, AI’s moderating effect on outcomes remains negative—highlighting the key distinction between quantity and quality of knowledge exchange. Therefore, while AI clearly expands knowledge-sharing capabilities, a critical question remains: what is the quality and strategic relevance of the knowledge being shared? AI-driven systems that prioritize efficiency and standardization over depth and contextual nuance may inadvertently limit Open Innovation Networks in generating truly innovative outcomes. When AI facilitates knowledge dissemination without ensuring alignment with the organization’s strategic goals, it risks flooding teams with information that lacks actionable or innovative value [107]. To be effective, AI-driven knowledge-sharing must support—not replace—judgment-based, innovation-oriented decision-making.

**Fig 6:**
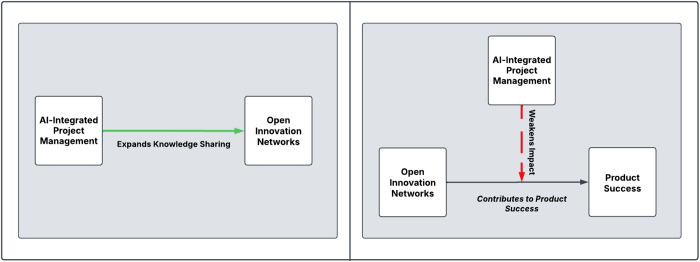
The dual role of AI in open innovation networks.

The dual role of AI is visually captured in Fig 6. The left side of the figure highlights AI’s enabling effect—its capacity to expand knowledge-sharing networks through more structured, accessible, and data-driven collaboration. This aligns with the statistical findings showing that higher levels of AI adoption are associated with more intensive and systematic knowledge-sharing practices. However, the right side of the figure reveals a critical tension: while AI improves knowledge structuring, it may also weaken the actual impact of Open Innovation Networks on product success. This negative moderation likely stems from the overformalization and rigidity introduced by AI tools that prioritize efficiency over exploration. For managers, this underscores the importance of balancing AI-driven systems with human flexibility and contextual judgment. AI should be strategically deployed to support collaborative workflows—not dominate them—especially in phases of product development where creativity, trust, and emergent insights are essential. Organizations are advised to adopt hybrid approaches that allow AI to augment, rather than replace, the social dynamics that drive innovation.

### Implications and future considerations

#### Theoretical implications.

The findings of this study contribute to the growing body of research on digital product success, particularly regarding the role of management strategies and AI-Integrated Project Management in shaping innovation outcomes. The study’s regression analysis demonstrates that Customer-Driven Product Development together with Open Innovation Networks and Organizational Digital Agility drive Digital Product Success and together account for 80.4% of its variance where Customer-Driven Product Development shows the highest impact. The findings support existing frameworks which identify customer-centric innovation and open collaboration together with digital adaptability as essential elements for successful product development [108]. The substantial explanatory power of the model strengthens the claim that sustained innovation requires firms to focus on customer engagement, external knowledge-sharing, and digital resilience during product development [109,110].

The study also adds to the ongoing debate on AI’s impact in digital product innovation. Although AI is frequently credited with enhancing efficiency and decision-making capabilities, research shows its direct contribution to Digital Product Success is minimal (p = 0.051) indicating that AI performance is dependent on specific circumstances [111]. The research uncovers that AI negatively influences the connection between Open Innovation Networks and Digital Product Success which provides new insights into how AI-enabled knowledge-sharing does not consistently result in better product performance. The discovery contradicts the belief that greater knowledge flow produces superior innovation results and emphasizes the importance of evaluating the quality and relevance of AI-generated insights.

#### Practical implications for digital product management.

For organizations aiming to enhance Digital Product Success, these findings provide critical insights into the key management factors that drive product success, their predictive power, and the role of AI in shaping innovation dynamics.

The leading predictive factor was Customer-Driven Product Development which confirmed the need for product development procedures to match customer requirements. To achieve optimal product-market fit and adoption rates organizations need to integrate agile methodologies together with iterative prototyping as well as continuous user feedback loops into their operational workflows [112]. The outcomes further demonstrate how Open Innovation Networks contribute to successful digital product development. Organizations gain access to new insights and technological advancements through external knowledge-sharing and collaboration but must carefully manage AI roles within these networks. Knowledge-sharing processes require AI to function as a supportive tool alongside human collaboration and not as a replacement to maintain decision-making efficiency [113]. The findings also highlight Organizational Digital Agility as essential for maintaining competitive advantage. Technologically adaptable organizations show superior ability to handle technological disruptions and employ innovative tools to sustain ongoing innovation. To remain agile in rapidly changing markets organizations must invest in scalable digital systems alongside AI automation tools and establish data-driven decision-making processes [114]. Organizations must carefully implement digital transformation strategies for seamless innovation processes.

The potential advantages of AI-Integrated Project Management can only be realized through strategic management. AI’s effectiveness as a tool for digital product success varies based on context while organizations need to maintain human-led project management practices with AI serving as supportive technology. Organizations should adopt AI in hybrid models that use AI to assist human experts in innovation processes instead of replacing them with fully autonomous decision-making systems [113,115].

Ultimately, success in AI integration for digital product management depends on organizations maintaining equilibrium between automated processes and human strategic decisions which enable AI to expand innovation rather than limit it.

### Limitations and future research

This study offers valuable insights into how AI-Integrated Project Management interacts with key organizational capabilities to influence digital product success. However, several limitations must be acknowledged, each offering promising directions for future research.

First, future studies should examine AI maturity levels—such as experimental use, rule-based systems, predictive models, and generative AI—as potential moderators in the relationships between Open Innovation Networks, Organizational Digital Agility, and Customer-Driven Product Development. This would provide a more nuanced understanding of how varying levels of AI sophistication influence innovation outcomes. Given the observed negative moderation effect between AI and Open Innovation Networks, further investigation is needed to determine whether more advanced or flexible AI implementations can mitigate this constraint and foster collaborative innovation.

In addition to AI maturity, the study’s sample composition presents another limitation. The data primarily reflect perspectives from European mid-sized and large firms, which may restrict generalizability to other regions or organizational types. Although subgroup analysis with non-European respondents confirmed the main relationships, future studies should aim for more geographically diverse samples, including startups and respondents from underrepresented regions such as Asia-Pacific, Africa, and North America. Where broader inclusion is not feasible, techniques like stratified sampling or weighted regression could help address potential regional bias.

Methodologically, the study conceptualized AI solely as a moderator. However, AI adoption and effectiveness may themselves be shaped by organizational capabilities, suggesting that AI could also be modeled as a dependent or moderated variable. Future research should consider reciprocal or cross-lagged modeling approaches to explore bidirectional relationships. For example, longitudinal studies could examine how innovation networks influence AI implementation strategies over time, and vice versa. Additionally, multi-level interaction models—such as three-way interactions—could reveal whether AI’s moderating role depends on other organizational or contextual factors.

Another limitation lies in the use of self-reported data from single respondents per organization, which introduces potential biases such as overestimation or social desirability. The anonymous and cross-sectional nature of the survey prevented the collection of objective performance indicators (e.g., ROI, adoption rates, customer satisfaction) or implementation of a multi-respondent design. To enhance data validity, future research should triangulate survey data with external performance metrics and gather inputs from multiple stakeholders within the same organization.

Conceptually, future studies could explore whether the quality of knowledge sharing mediates the relationship between Open Innovation Networks and product success, especially in AI-supported environments. While AI was found to increase the structure and frequency of knowledge exchange, its influence on the novelty or usefulness of shared knowledge remains unclear. Testing AI-generated knowledge quality as a mediator could offer deeper insight into innovation performance mechanisms. Industry-level comparative analyses (e.g., by digital maturity or company size) may further reveal whether AI’s effects are contingent on sector-specific conditions or resource availability.

While this study captured a snapshot of AI’s role in digital innovation, its cross-sectional design limited the ability to draw causal inferences or observe how AI’s influence evolves throughout the digital product lifecycle. Future research should employ longitudinal or panel-based designs to track the trajectory of AI integration across key phases—ideation, development, launch, and post-launch learning. Such designs would help uncover time-lagged effects and feedback loops and clarify whether early AI implementation produces cumulative benefits or phase-dependent shifts in innovation performance.

Finally, incorporating socio-technical factors such as employee trust in AI systems, organizational AI readiness, and innovation culture could deepen our understanding of when and how AI integration succeeds. These variables may condition the effectiveness of AI beyond its technical configuration. Investigating hybrid project models—where AI augments rather than replaces human decision-making—could also reveal optimal setups for sustained innovation.

Together, these directions offer a path toward a more comprehensive and dynamic understanding of AI’s multifaceted role in shaping innovation and product success across diverse organizational contexts.

## Conclusion

This study set out to investigate how Customer-Driven Product Development, Open Innovation Networks, Organizational Digital Agility, and AI-Integrated Project Management contribute to Digital Product Success. Through regression and moderation analyses, the findings confirmed that the first three factors have significant positive effects on product success, while AI-Integrated Project Management showed only a marginal direct effect. Notably, AI negatively moderated the relationship between Open Innovation Networks and Product Success, revealing that while AI can support structured knowledge-sharing, it may weaken collaborative innovation if not thoughtfully applied.

The results contribute to the theoretical discourse by affirming the critical role of customer-centric development, open collaboration, and organizational agility in driving successful digital products. On the other hand, this research contradicts the common belief that AI exclusively benefits innovation management by demonstrating that AI’s usefulness varies based on its application context. Interestingly, although higher levels of AI adoption expand knowledge-sharing practices—which are foundational for building strong Open Innovation Networks—this enhancement does not necessarily translate into greater product success. Instead, the presence of AI can dampen the impact of open innovation on outcomes, indicating a tension between structure and creativity in digitally mediated collaboration.

For practitioners the research results highlight the importance to emphasize customer input together with cross-functional knowledge sharing and organizational flexibility when developing digital products. While AI tools can enhance operational processes and support the organization of knowledge, their impact on innovation outcomes is not inherently positive. Organizations need to implement hybrid systems where AI functions as a support tool to human creativity rather than taking its place, especially in collaborative scenarios demanding trust and flexible knowledge sharing.

Future research should further examine AI’s impact on decision-making, knowledge-sharing quality, and its interaction with human expertise in product development. It is also important to explore how AI functions across different industries and stages of the product lifecycle, as its effectiveness appears highly context-dependent. Identifying optimal strategies for balancing the quantity and quality of AI-driven knowledge-sharing will be key to ensuring that AI supports, rather than constrains, innovation in digital product ecosystems. Additionally, to better understand why AI weakens the relationship between Open Innovation Networks and product success, future research should employ qualitative or mixed-method designs such as interviews or content analysis. While this study offers theoretical explanations using the STS, DOI, and TOE frameworks, it does not capture the interpersonal and contextual dynamics behind this effect. Qualitative insights could reveal how AI impacts trust, collaboration, or creativity in open innovation settings—factors not easily observed through surveys.

Ultimately, this study reinforces that Digital Product Success is best achieved through a balanced interplay of technology and human-driven strategies. While AI offers undeniable advantages, its implementation must be deliberate and context-sensitive. The most resilient and innovative organizations will be those that not only invest in digital capabilities but also preserve the collaborative and adaptive qualities that underpin successful product development.

## Supporting information

S1 File Regression and moderation analyses results. Calculated by the author: Conducted using SPSS software version 30.0.
(PDF)

S2 File Survey questions. (PDF)

S3 File Constructs operationalization. (PDF)
