# Supplementary material for: Digital product success under the microscope: When artificial intelligence in projects helps — and when it hurts
Source: PLoS One. 2025 Aug 29;20(8):e0331229. doi: 10.1371/journal.pone.0331229 (PMC12396679; doi:10.1371/journal.pone.0331229)
Supplement: S1 File — Calculated by the author: Conducted using SPSS software version 30.0. (PDF) [file pone.0331229.s001.pdf]

Full Regression Model

Variables Entered/Removed[a]

Model	Variables Entered	Variables Removed	Method
1	Cavg, Aavg, davg, Kavg[b]	.	Enter

a. Dependent Variable: Pavg

b. All requested variables entered.

Model Summary[b]

Model	R	R Square	Adjusted R Square	Std. Error of the Estimate
1	.897[a]	.804	.801	.40014

a. Predictors: (Constant), Cavg, Aavg, davg, Kavg

b. Dependent Variable: Pavg

ANOVA[a]

Model		Sum of Squares	df	Mean Square	F	Sig.
1	Regression	153.836	4	38.459	240.196	.000[b]
	Residual	37.467	234	.160		
	Total	191.303	238			

a. Dependent Variable: Pavg

b. Predictors: (Constant), Cavg, Aavg, davg, Kavg

Coefficients[a]

		Unstandardized Coefficients		Standardized Coefficients		
Model		B	Std. Error	Beta	t	Sig.
1	(Constant)	.165	.115		1.432	.154
	Kavg	.166	.040	.179	4.162	.000
	Aavg	.073	.037	.067	1.964	.051
	davg	.146	.038	.145	3.826	.000
	Cavg	.586	.043	.636	13.678	.000

a. Dependent Variable: Pavg

Residuals Statistics[a]

	Minimum	Maximum	Mean	Std. Deviation	N
Predicted Value	1.3838	4.7072	3.2897	.80397	239
Residual	-1.22169	1.46169	.00000	.39677	239
Std. Predicted Value	-2.371	1.763	.000	1.000	239
Std. Residual	-3.053	3.653	.000	.992	239

a. Dependent Variable: Pavg

Moderation

Regression 1 – AI as moderator between organizational digital agility and product success

Variables Entered/Removed[a]

Model	Variables Entered	Variables Removed	Method
1	AavgxDavg, davg, Aavg[b]	.	Enter

a. Dependent Variable: Pavg

b. All requested variables entered.

Model Summary[b]

Model	R	R Square	Adjusted R Square	Std. Error of the Estimate
1	.627[a]	.393	.385	.70294

a. Predictors: (Constant), AavgxDavg, davg, Aavg

b. Dependent Variable: Pavg

ANOVA[a]

Model		Sum of Squares	df	Mean Square	F	Sig.
1	Regression	75.185	3	25.062	50.719	.000[b]
	Residual	116.118	235	.494		
	Total	191.303	238			

a. Dependent Variable: Pavg

b. Predictors: (Constant), AavgxDavg, davg, Aavg

Coefficients[a]

Model		Unstandardized Coefficients B	Std. Error	Standardized Coefficients Beta	t	Sig.
1	(Constant)	1.141	.562		2.029	.044
	Aavg	.169	.249	.157	.680	.497
	davg	.504	.162	.499	3.107	.002
	AavgxDavg	.007	.066	.034	.102	.919

a. Dependent Variable: Pavg

Residuals Statistics[a]

	Minimum	Maximum	Mean	Std. Deviation	N
Predicted Value	2.0070	4.4934	3.2897	.56205	239
Residual	-1.85576	2.14423	.00000	.69849	239
Std. Predicted Value	-2.282	2.142	.000	1.000	239
Std. Residual	-2.640	3.050	.000	.994	239

a. Dependent Variable: Pavg

Regression 2 – AI as moderator between customer-driven product development and product success

Variables Entered/Removed[a]

Model	Variables Entered	Variables Removed	Method
1	AavgxCavg, Cavg, Aavg[b]	.	Enter

a. Dependent Variable: Pavg

b. All requested variables entered.

Model Summary[b]

Model	R	R Square	Adjusted R Square	Std. Error of the Estimate
1	.883[a]	.780	.778	.42285

a. Predictors: (Constant), AavgxCavg, Cavg, Aavg

b. Dependent Variable: Pavg

ANOVA[a]

Model		Sum of Squares	df	Mean Square	F	Sig.
1	Regression	149.284	3	49.761	278.297	.000[b]
	Residual	42.019	235	.179		
	Total	191.303	238			

a. Dependent Variable: Pavg

b. Predictors: (Constant), AavgxCavg, Cavg, Aavg

Coefficients[a]

Model		Unstandardized Coefficients		Standardized Coefficients	t	Sig.
		B	Std. Error	Beta		
1	(Constant)	-.041	.301		-.137	.891
	Aavg	.355	.128	.329	2.780	.006
	Cavg	.901	.088	.978	10.238	.000
	AavgxCavg	-.061	.035	-.298	-1.733	.084

a. Dependent Variable: Pavg

Residuals Statistics[a]

	Minimum	Maximum	Mean	Std. Deviation	N
Predicted Value	1.3650	4.6461	3.2897	.79199	239
Residual	-1.36141	1.42027	.00000	.42018	239
Std. Predicted Value	-2.430	1.713	.000	1.000	239
Std. Residual	-3.220	3.359	.000	.994	239

a. Dependent Variable: Pavg

Regression 3 - AI as moderator between open innovation networks and product success

Variables Entered/Removed[a]

Model	Variables Entered	Variables Removed	Method
1	KavgXAavg, Kavg, Aavg[b]	.	Enter

a. Dependent Variable: Pavg

b. All requested variables entered.

Model Summary[b]

Model	R	R Square	Adjusted R Square	Std. Error of the Estimate
1	.773[a]	.598	.593	.57189

a. Predictors: (Constant), KavgXAavg, Kavg, Aavg

b. Dependent Variable: Pavg

ANOVA[a]

Model		Sum of Squares	df	Mean Square	F	Sig.
1	Regression	114.445	3	38.148	116.641	.000[b]
	Residual	76.858	235	.327		
	Total	191.303	238			

a. Dependent Variable: Pavg

b. Predictors: (Constant), KavgXAavg, Kavg, Aavg

Coefficients[a]

Model		Unstandardized Coefficients		Standardized Coefficients	t	Sig.
		B	Std. Error	Beta		
1	(Constant)	-.460	.378		-1.219	.224
	Aavg	.702	.145	.651	4.836	.000
	Kavg	.984	.120	1.059	8.220	.000
	KavgXAavg	-.144	.043	-.693	-3.343	.001

a. Dependent Variable: Pavg

Residuals Statistics[a]

	Minimum	Maximum	Mean	Std. Deviation	N
Predicted Value	1.5316	4.3601	3.2897	.69344	239
Residual	-1.72703	2.09139	.00000	.56827	239
Std. Predicted Value	-2.535	1.544	.000	1.000	239
Std. Residual	-3.020	3.657	.000	.994	239

a. Dependent Variable: Pavg
