# Supplementary material for: Digital product success under the microscope: When artificial intelligence in projects helps — and when it hurts
Source: PLoS One. 2025 Aug 29;20(8):e0331229. doi: 10.1371/journal.pone.0331229 (PMC12396679; doi:10.1371/journal.pone.0331229)
Supplement: S2 File — (PDF) [file pone.0331229.s002.pdf]

Socio-Demographic Information

Age

1. Please specify your age:
- 18-24
- 25-34
- 35-44
- 45-54
- 55-64
- 65 and above

Gender

2. What is your gender?
- Male
- Female
- Prefer not to say

Education Level

3. What is the highest level of education you have completed?
- High school or equivalent
- Associate degree
- Bachelor’s degree
- Master’s degree
- Doctorate (PhD)
- Other (please specify): ________________

Years of Experience

4. How many years of professional experience do you have?
- Less than 1 year
- 1-3 years
- 4-6 years
- 7-10 years
- More than 10 years

Job Position

5. What is your current job position?
- Intern
- Entry-level
- Mid-level

- Senior-level
- Managerial
- Executive
- Other (please specify): _______________

Industry

6. Which industry do you work in?

- Information Technology
- Finance
- Healthcare
- Manufacturing
- Education
- Retail
- Consulting
- Government
- Other (please specify): _______________

Company Size

7. What is the size of your company?
    - 1-10 employees
    - 11-50 employees
    - 51-200 employees
    - 201-500 employees
    - 501-1000 employees
    - More than 1000 employees

Geographic Location

8. In which region is your company primarily located?
    - North America
    - South America
    - Europe
    - Africa
    - Asia
    - Australia

Department

9. Which department do you work in?
    - Research & Development
    - Sales & Marketing

  - Human Resources
  - Finance & Accounting
  - Operations
  - IT & Services
- Other (please specify): _______________

Employment Status

10. What is your current employment status?
- Full-time
- Part-time
- Self-employed
- Unemployed
- Student
- Retired

Work Location

11. Where do you primarily work?
    - On-site
    - Remote
    - Hybrid (mix of on-site and remote)

Company Type

12. What type of company do you work for?
- Private
- Public
- Non-profit
- Government

<u>Instructions:</u>

The following sections contain statements related to various aspects of organizational practices. Please rate your level of agreement with each statement on a scale of 1 to 5, where 1 indicates strong disagreement and 5 indicates strong agreement.

Open Innovation Networks

Knowledge Co-Creation

1. Our organization actively engages in co-creating knowledge with external partners (e.g., suppliers, customers, research institutions).
2. I frequently participate in joint innovation projects with external entities.

3. Our organization's efforts to leverage external knowledge sources for innovation are very effective.
4. Customer feedback is thoroughly integrated into our product development process.

Knowledge Sharing and Distribution

1. Employees frequently share knowledge with both internal and external stakeholders.
2. Our organization encourages the distribution of knowledge across organizational boundaries.
3. The knowledge distribution practices in our organization are highly effective.
4. Knowledge-sharing practices significantly improve the overall innovation capacity of our organization.

Collaborative Technologies Integration

1. Our organization extensively uses collaborative technologies (e.g., cloud-based platforms, social media) to support knowledge sharing.
2. Collaborative technologies are well integrated into our organization's workflows.
3. Employees frequently use collaborative technologies for project management and communication.
4. The collaborative technologies used in our organization significantly enhance productivity.

AI-Integrated Project Management

AI Utilization

1. Our organization frequently uses AI tools in project management.
2. AI contributes significantly to project decision-making.
3. I regularly use AI tools in my daily project management tasks.
4. AI tools are well integrated into our project management software.

AI Effectiveness

1. AI tools are very effective in improving project planning accuracy.
2. AI-driven insights are extremely helpful in managing project risks.
3. AI has greatly improved project forecasting and budgeting accuracy.
4. AI tools frequently contribute to successful project outcomes.

AI Integration

1. AI tools are well integrated with our existing project management systems.
2. AI has significantly improved our project management processes.
3. The AI tools used in our organization are very user-friendly.
4. AI tools provide actionable insights for project management to a great extent.

Organizational Digital Agility

Digital Adaptation

1. Our organization quickly adapts to new digital tools and technologies.
2. We have effective strategies in place to manage digital disruptions.
3. Employees are trained to handle digital transitions and disruptions effectively.
4. We frequently review and improve our digital adaptation processes.

Cybersecurity and Risk Management

1. Our organization has robust cybersecurity measures in place.
2. We conduct regular cybersecurity training for all employees.
3. Our organization has effective protocols to respond to cyber-attacks.
4. Our cybersecurity practices significantly reduce the risk of digital threats.

Digital Recovery and Continuity

1. Our organization has a comprehensive digital recovery plan.
2. We can quickly restore digital services after disruptions.
3. Our organization regularly tests its digital recovery procedures.
4. Our digital recovery practices ensure minimal disruption to business operations.

Customer-Driven Product Development

Customer Involvement in Innovation

1. Our organization actively involves customers in the product development process.
2. Customer feedback is regularly incorporated into our product designs.
3. We frequently collaborate with customers to identify their needs and preferences.
4. Our organization values and acts upon customer suggestions and ideas.

Market Research and Analysis

1. We conduct regular market research to understand customer needs and trends.
2. Our organization frequently analyzes customer feedback to improve products.
3. Customer satisfaction metrics are a key component of our product development process.
4. We actively monitor customer feedback on various channels to gauge product performance.

Product Customization and Personalization

1. Our products are designed to meet the specific needs of different customer segments.
2. We offer personalized product options based on customer preferences.
3. Our organization regularly updates products to better match customer expectations.
4. Our product offerings reflect a deep understanding of customer personalization needs.

Digital Product Success

Product Performance

1. The products developed by our organization in the last year have been very successful.
2. Our products meet customer expectations to a high degree.
3. I am very satisfied with the quality of the products developed by our organization.
4. Our products frequently receive positive feedback from customers.

Market Performance

1. Our products have been more successful in the market compared to competitors.
2. The profitability of our new products is very high.
3. Our products consistently achieve their sales targets.
4. Our products gain market share over time to a significant extent.

Innovation and Quality

1. The products developed by our organization are very innovative.
2. Our products maintain high-quality standards to a great extent.
3. New product features are frequently developed and released.
4. Customer satisfaction with the innovation in our products is very high.
