# Supplementary material for: Digital product success under the microscope: When artificial intelligence in projects helps — and when it hurts
Source: PLoS One. 2025 Aug 29;20(8):e0331229. doi: 10.1371/journal.pone.0331229 (PMC12396679; doi:10.1371/journal.pone.0331229)
Supplement: S3 File — (PDF) [file pone.0331229.s003.pdf]

| Open Innovation Networks | |
|---|---|
| **Subdimension: Knowledge Co-Creation** | |
| Our organization actively engages in co-creating knowledge with external partners (e.g., suppliers, customers, research institutions). | [57] [58] |
| I frequently participate in joint innovation projects with external entities. | |
| Our organization's efforts to leverage external knowledge sources for innovation are very effective. | |
| Customer feedback is thoroughly integrated into our product development process. | |
| **Subdimension: Knowledge Sharing and Distribution** | |
| Employees frequently share knowledge with both internal and external stakeholders. | [59] [60] |
| Our organization encourages the distribution of knowledge across organizational boundaries. | |
| The knowledge distribution practices in our organization are highly effective. | |
| Knowledge-sharing practices significantly improve the overall innovation capacity of our organization. | |
| **Subdimension: Collaborative Technologies Integration** | |
| Our organization extensively uses collaborative technologies (e.g., cloud-based platforms, social media) to support knowledge sharing. | [61] [62] |
| Collaborative technologies are well integrated into our organization's workflows. | |
| Employees frequently use collaborative technologies for project management and communication. | |
| The collaborative technologies used in our organization significantly enhance productivity. | |
| **AI-Integrated Project Management** | |
| **Subdimension: AI Utilization** | |
| Our organization frequently uses AI tools in project management. | [63] [64] |
| AI contributes significantly to project decision-making. | |
| I regularly use AI tools in my daily project management tasks. | |
| AI tools are well integrated into our project management software. | |
| **Subdimension: AI Effectiveness** | |
| AI tools are very effective in improving project planning accuracy. | [65] [66] |
| AI-driven insights are extremely helpful in managing project risks. | |
| AI has greatly improved project forecasting and budgeting accuracy. | |
| AI tools frequently contribute to successful project | |

| | |
|---|---|
| outcomes. | |
| **Subdimension: AI Integration** | |
| AI tools are well integrated with our existing project management systems. | [67] [68] |
| AI has significantly improved our project management processes. | |
| The AI tools used in our organization are very user-friendly. | |
| AI tools provide actionable insights for project management to a great extent. | |
| **Organizational Digital Agility** | |
| **Subdimension: Digital Adaptation** | |
| Our organization quickly adapts to new digital tools and technologies. | [69] [70] |
| We have effective strategies in place to manage digital disruptions. | |
| Employees are trained to handle digital transitions and disruptions effectively. | |
| We frequently review and improve our digital adaptation processes. | |
| **Subdimension: Cybersecurity and Risk Management** | |
| Our organization has robust cybersecurity measures in place. | [71] [72] |
| We conduct regular cybersecurity training for all employees. | |
| Our organization has effective protocols to respond to cyber-attacks. | |
| Our cybersecurity practices significantly reduce the risk of digital threats. | |
| **Subdimension: Digital Recovery and Continuity** | |
| Our organization has a comprehensive digital recovery plan. | [73] [74] |
| We can quickly restore digital services after disruptions. | |
| Our organization regularly tests its digital recovery procedures. | |
| Our digital recovery practices ensure minimal disruption to business operations. | |
| **Customer-Driven Product Development** | |
| **Subdimension: Customer Involvement in Innovation** | |
| Our organization actively involves customers in the product development process. | [75] [76] |
| Customer feedback is regularly incorporated into our product designs. | |
| We frequently collaborate with customers to identify their needs and preferences. | |
| Our organization values and acts upon customer suggestions and ideas. | |

| | |
|---|---|
| **Subdimension: Market Research and Analysis** | |
| We conduct regular market research to understand customer needs and trends. | [77]<br>[78] |
| Our organization frequently analyzes customer feedback to improve products. | |
| Customer satisfaction metrics are a key component of our product development process. | |
| We actively monitor customer feedback on various channels to gauge product performance. | |
| **Subdimension: Product Customization and Personalization** | |
| Our products are designed to meet the specific needs of different customer segments. | [79]<br>[80] |
| We offer personalized product options based on customer preferences. | |
| Our organization regularly updates products to better match customer expectations. | |
| Our product offerings reflect a deep understanding of customer personalization needs. | |
| **Digital Product Success** | |
| **Subdimension: Product Performance** | |
| The products developed by our organization in the last year have been very successful. | [8]<br>[81] |
| Our products meet customer expectations to a high degree. | |
| I am very satisfied with the quality of the products developed by our organization. | |
| Our products frequently receive positive feedback from customers. | |
| **Subdimension: Market Performance** | |
| Our products have been more successful in the market compared to competitors. | [82]<br>[83] |
| The profitability of our new products is very high. | |
| Our products consistently achieve their sales targets. | |
| Our products gain market share over time to a significant extent. | |
| **Subdimension: Innovation and Quality** | |
| The products developed by our organization are very innovative. | [84]<br>[85] |
| Our products maintain high-quality standards to a great extent. | |
| New product features are frequently developed and released. | |
| Customer satisfaction with the innovation in our products is very high. | |
